# Frying

**Published:** 1886-01

**Authors:** 


**Frying.**—Fried meats are, of all, the most objectionable, owing to their comparative indigestibility, due in great measure to the chemical change which the fat employed undergoes in the operation. If the meat is very juicy it will not fry well because it becomes sodden before the water is evaporated, and it will not brown because the temperature is too low to scorch it.

To fry fish well the fat should be boiling hot and the fish well dried in a cloth. Otherwise, owing to the generation of steam the temperature will fall so low that it will be boiling in *its own* steam and not be browned. Meat, or, indeed, any article should be frequently turned or agitated while frying to promote the evaporation of the watery particles To make fried things look well, they should be done over twice with egg and stale bread crumbs.

